# MyoNet: Deep Learning-Based Myocardial Strain Quantification from Cine Cardiac MRI

**DOI:** 10.3390/bioengineering13030310

**Published:** 2026-03-07

**Authors:** Dayeong An, Andrew Nencka, Patrick Clarysse, Pierre Croisille, Carmen Bergom, El-Sayed Ibrahim

**Affiliations:** 1 Department of Radiology, Northwestern University, Chicago, IL 60611, USA; dayeong.an@northwestern.edu; 2 Department of Radiology, Medical College of Wisconsin, Milwaukee, WI 53226, USA; anencka@mcw.edu; 3 CREATIS, 69621 Lyon, France; 4 Department of Radiation Oncology, Washington University, St. Louis, MO 63110, USA

**Keywords:** cine cardiac MRI, myocardial strain, regional myocardial function, cardiac motion estimation, CMR tagging, circumferential strain, radial strain, deep learning, cardiac dysfunction

## Abstract

To develop and assess MyoNet, a deep learning (DL)-based network for measuring myocardial regional function from cine cardiac magnetic resonance (CMR) images, and compare its efficacy with ResMyoNet as an efficient alternative to SinMod-derived reference. MyoNet was tested alongside ResMyoNet on datasets from Dahl salt-sensitive rat models undergoing radiation therapy (RT). Both networks were designed to extract displacement maps from cine images, were specifically optimized for detailed myocardial deformation, employed advanced convolution operations with alternating kernel sizes for spatial and temporal analysis, and robust loss functions. MyoNet demonstrated superior performance in myocardial strain measurement, achieving high consistency with the SinMod-derived reference strains. It outperformed ResMyoNet, achieving higher performance metrics, including SSIM of 0.961 and 0.960, ICC of 0.973 and 0.975, and Pearson CC of 0.973 and 0.953 for circumferential (Ecc) and radial (Err) strains, respectively. Its accuracy and efficiency in generating strain measurements were validated through comprehensive statistical analyses. MyoNet offers a significant advancement in myocardial strain analysis from cine CMR images, potentially revolutionizing cardiac imaging in pre-clinical studies. Its ability to provide detailed and reliable measurements positions it as a valuable tool for clinical applications, particularly in monitoring the cardiac health of cancer patients.

## 1. Introduction

The significance of regional cardiac function analysis, especially using cardiac resonance (CMR) myocardial strain analysis, has gained increased attention. Myocardial strain allows for a detailed understanding of myocardial contractility compared to conventional global function measures. Traditionally, the evaluation of cardiac function has mainly relied on measuring left ventricular ejection fraction (LVEF), a valuable yet limited tool that primarily provides global information about cardiac function without the ability to detect localized myocardial contractility abnormalities or dysfunctions [1]. Therefore, LVEF can mask underlying subclinical cardiac dysfunction, thus delaying the detection of heart diseases, as in thoracic radiation-induced cardiotoxicity [1,2]. Radiation therapy (RT), essential in cancer treatment, can inadvertently damage the heart, leading to long-term complications including ischemic heart disease, fibrosis, arrhythmias, cardiomyopathy, valvular abnormalities, and pericarditis, particularly in breast and lung cancer treatments [3,4,5,6,7]. These complications often emerge subclinically, making early detection crucial [6,8].

Animal models, such as Dahl salt-sensitive (SS) rats, provide valuable insights into cardiotoxic implications post-RT [9,10], helping understand disease progression and potential interventions. Cardiac magnetic resonance (CMR) has emerged as integral in measuring regional cardiac function, offering non-invasive evaluation without ionizing radiation. CMR sequences, like tagging [11] and CMR feature tracking (CMR-FT) [12], have been developed for this purpose. However, CMR tagging is not widely adopted in clinical practice due to the necessary longer acquisition times and the need for special post-processing techniques [13,14,15], while CMR-FT lacks intramyocardial markers, affecting accuracy in regional strain analysis [16,17]. These challenges are exacerbated in small animal models due to differences in image contrast and magnetic field strengths in pre-clinical and clinical MRI studies [18,19].

In this study, we propose the integration of deep learning (DL) with cine CMR to generate fast, accurate, and reproducible myocardial strain analysis without the need for tagging. Specifically, we use supervised learning to allow DL models to learn from tagged examples and predict strain from cine images. By leveraging the detailed displacement fields from the tagged images, our goal is to design, train, and compare two DL networks, MyoNet and ResMyoNet, to create an accurate, rapid algorithm for generating myocardial strain measurements from routinely acquired cine images. We hypothesize that these networks will allow for effective and efficient estimation of myocardial strain, therefore providing innovative solutions for early detection of subclinical cardiac function in cardiotoxicity.

## 2. Materials and Methods

### 2.1. Study Population and Data Preprocessing

**Figure 1:**
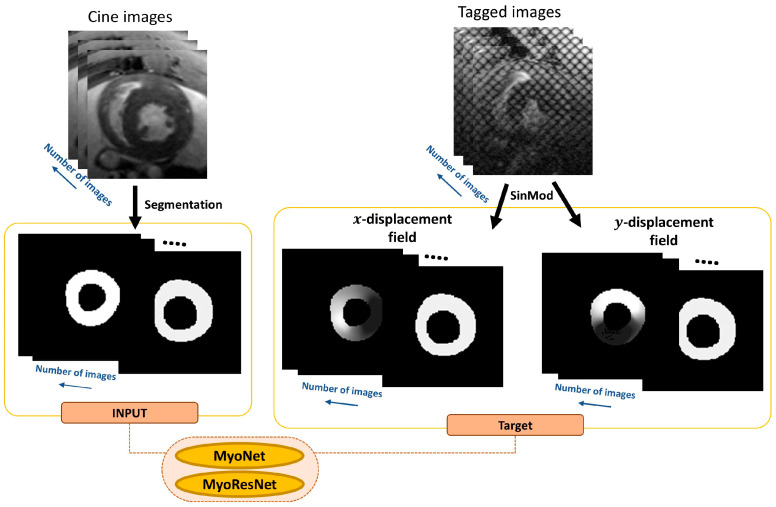
Workflow of the image processing and deep learning framework. Cine and tagged MRI images are shown on the top. Cine images are then segmented into binary images to be used as input. Concurrently, tagged images undergo SinMod to generate x- and y-displacement fields, which are utilized as target datasets for the networks MyoNet and ResMyoNet.

This study (Figure 1) was approved by the institutional animal care committee of the Medical College of Wisconsin and used data from 22 Dahl SS rats undergoing RT, yielding 64 short-axis slices, each containing 20 cine and corresponding tagged timeframes of cardiac MRI images, as previously described [20,21]. The initial preprocessing involved a manual segmentation of the left ventricle (LV) in cine images to highlight the LV myocardium by identifying the endocardium and epicardium contours to generate binary images. All images were then cropped to uniform dimensions of 128 × 128.

For tagged images, the SinMod technique (InTag, Lyon, France) [14,22] was utilized to generate x- and y-displacement fields, which served as the reference standard (ground truth) for network training. These displacement fields were aligned with the segmented cine images and rescaled to an intensity range between 32 and 255 for clear differentiation of the image background (set to 0). This scaling was an invertible affine normalization used only to bound network outputs. Predicted values were transformed back to signed displacement in the original units using the inverse affine mapping, and all displacement error metrics and strain calculations were performed after this inverse transform. These displacement fields were also standardized to dimensions of 128 × 128 through cropping to match cine images.

### 2.2. MyoNet

MyoNet is a novel deep learning network tailored for myocardial strain analysis using spatiotemporal data in CMR. It utilizes the capabilities of DL for processing spatial (in-slice tissue motion) and temporal (tissue motion across consecutive timeframes) dimensions, aiming to improve the accuracy and efficiency of strain analysis.

**Figure 2:**
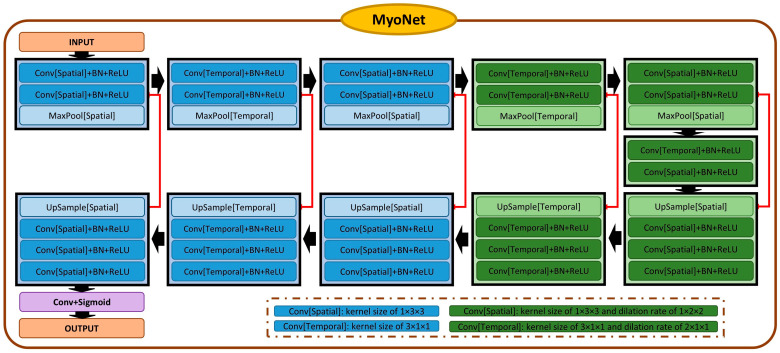
Schematic representation of the MyoNet. The network is structured similarly to Unet, consisting of five encoding and decoding layers. Input is segmented binary cine spatiotemporal images, and output is segmented x- and y-displacement fields.

MyoNet, similar to the conventional Unet [23,24] (Figure 2), includes five encoding and decoding layers, capturing both local features through the down-sampling path (encoder) and larger context through the up-sampling path (decoder).

In the encoding stage, MyoNet employs sequential 3D convolution operations followed by a max pooling operation with alternating kernel sizes (1 × 3 × 3 and 3 × 1 × 1) for distinct spatial and temporal feature capture. Dilation in deeper layers, combined with varied kernel sizes and alternating dilation rates (1 × 2 × 2 and 2 × 1 × 1), expands the receptive field, allowing global feature learning across a wider spatiotemporal extent without compromising resolution. The decoder, symmetrical to the encoder, uses up-sampling and convolutional blocks, enriched by skip connections to preserve high-frequency details. The network concludes with a 1 × 1 × 1 convolution and a sigmoid activation function, normalizing the output [25].

MyoNet is optimized for straightforward and efficient feature extraction, minimizing overfitting and enhancing interpretability. Its architecture focuses on context understanding within the data, crucial for myocardial strain analysis.

### 2.3. ResMyoNet

**Figure 3:**
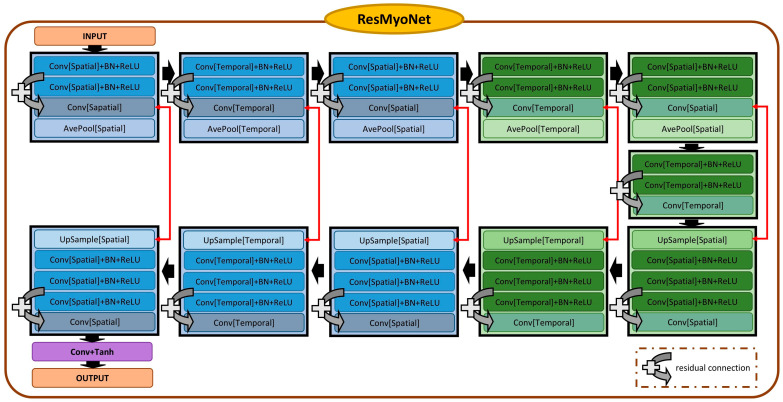
Schematic representation of the ResMyoNet. The network is structured similarly to ResUnet, consisting of five encoding and decoding layers. Input and output specifications align with those of MyoNet. The primary distinction is the block composition, emphasized by the integration of residual connections.

ResMyoNet exhibits significant similarities to MyoNet, especially when handling spatiotemporal data (Figure 3), and distinguishes itself with its modified ResUnet architecture [26]. It incorporates residual connections for efficient gradient flow during training. It employs a series of layers with custom residual blocks, each comprising a shortcut path and a main path with spatiotemporal convolutions, followed by batch normalization and ReLU activation. The tanh activation function is applied post-convolution, aiding in gradient propagation and training stability [27,28].

ResMyoNet’s residual connections not only facilitate faster convergence and mitigate the vanishing gradient problem but also contribute to its ability for deeper and more intricate feature extraction.

### 2.4. Experimental Setup

Our dataset included 64 short-axis slices from 22 scans, each comprising 20 cine images and corresponding SinMod-derived x- and y-displacement fields. The input data were standardized by normalizing to ensure consistency for the network’s learning process. Additionally, data augmentation techniques were applied to enhance diversity and assist in model generalization. The dataset was divided at the scan level (to prevent data leakage between sets) into distinct training (80%), validation (10%), and testing sets (10%). The training set underwent further data augmentation through random flip techniques to enhance the model’s robustness and adaptability. The validation set was crucial for hyperparameter tuning and performance evaluation, while the testing set served as the final model assessment on unseen data.

Model implementation was conducted using PyTorch version 2.0.1 and Python 3.11 on a high-performance computing system equipped with an NVIDIA Quadro GV100 GPU. Training utilized the RMSprop optimizer with an initial learning rate of 1 × 10^−3^. The batch size was set to 4. Both networks were trained for 100 epochs, with model selection based on the best validation loss.

### 2.5. Optimization and Loss Functions

During the training, a combination of loss functions was utilized to effectively guide the optimization process. First, the mean squared error (MSE) loss was used to penalize large errors and improve accuracy in estimating x- and y-displacement fields. The MSE is defined as follows:

(1) $$L_{MSE}=\frac{1}{n}\sum_{i=1}^{n}{\left(x_{i}-\hat{x_{i}}\right)}^{2}{+\left(y_{i}-\hat{y_{i}}\right)}^{2},$$

Here $x_{i}$ and $y_{i}$ signify the normalized pixel values representing x- and y-displacement fields generated by tagging, while $\hat{x_{i}}$ and $\hat{y_{i}}$ are the predicted x- and y-displacement fields from our models at the time frame i and n is the number of frames. Simultaneously, the smooth L1 loss was introduced, functioning akin to the L2 loss for small differences and resembling the L1 loss for larger differences, which is formulated as follows:

(2) $$L_{Smooth}=\left\{\begin{array}{ll} 0.5\left[{\left(x_{ij}-\hat{x_{ij}}\right)}^{2}+{\left(y_{ij}-\hat{y_{ij}}\right)}^{2}\right] & if\;\left|x_{ij}-\hat{x_{ij}}\right|<1\;and\;\left|y_{ij}-\hat{y_{ij}}\right|<1\\ \left|x_{ij}-\hat{x_{ij}}\right|+\left|y_{ij}-\hat{y_{ij}}\right|-0.5 & otherwise \end{array}\right.,$$

Here j is the pixel location while i is the time frame. This combination fosters a balance that enhances robustness to outliers, maintaining the capacity to underscore significant inconsistencies. Beyond these, a specialized loss was crafted exclusively for this application. This custom loss emphasizes errors where the segmented areas of the x- and y-displacement fields were generated by tagging.

(3) $$L_{custom}=\left\{\begin{array}{ll} \frac{\sum_{j=1}^{m}z_{ij}}{m} & if\;m>0\\ 0 & otherwise \end{array}\right.\;z_{ij}=\left\{\begin{array}{ll} {\left(x_{ij}-\hat{x_{ij}}\right)}^{2}+{\left(y_{ij}-\hat{y_{ij}}\right)}^{2} & if\;x_{ij}\;and\;y_{ij}>0.125\\ 0 & otherwise \end{array}\right.,$$

As mentioned, the x- and y-displacement fields were rescaled to pixel values ranging from 32 to 255. After normalization, this range is transformed to 0.125 to 1.

The total loss function was computed as a combination of the three components:

(4) $$L_{Total}=L_{MSE}+L_{smooth}+L_{custom}$$

### 2.6. Statistical Analysis

The model performance of MyoNet and ResMyoNet was evaluated using several metrics. For displacement field assessment, we computed the structural similarity index (SSIM) [29] for image quality, root mean squared error (RMSE), and mean endpoint error (EPE) for vector displacement accuracy. Additionally, strains derived from MyoNet and ResMyoNet were compared with SinMod-derived reference values using the intraclass correlation coefficients (ICC) [30], Pearson correlation coefficient [31], and coefficient of variation (CV). To evaluate the agreement between strains acquired from MyoNet, ResMyoNet, and SinMod, a Bland–Altman plot analysis was also performed [32]. Training accuracy was defined as 1 minus the normalized mean squared error between predicted and target displacement fields.

### 2.7. Strain Computation

Circumferential (Ecc) and radial (Err) strains were computed from the predicted x- and y-displacement fields using a Lagrangian formulation. For each timeframe, the 2D displacement vector $u\left(x,y\right)=\left[u_{x}\left(x,y\right),\;u_{y}\left(x,y\right)\right]$ was converted to a deformation gradient F=I+∇u, where spatial gradients were calculated on the grid using central finite differences within the myocardium mask. The Green-Lagrange strain tensor was computed as $E=0.5\left(F^{T}F-I\right)$ [33]. For segment analysis, pixel-wise strains were averaged within each of the six AHA short-axis segments [34].

## 3. Results

### 3.1. Strain Analysis by MyoNet and ResMyoNet

**Figure 4:**
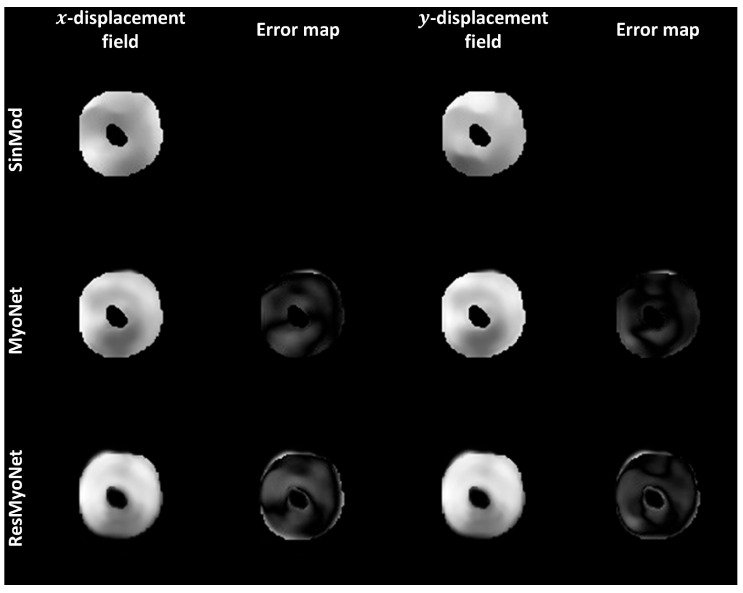
The figure displays x- and y-displacement field images derived from SinMod-derived reference, MyoNet, and ResMyoNet. Error maps highlight the discrepancies between displacement fields compared to SinMod. The error maps offer a detailed view of the spatial distribution of errors, emphasizing regions of higher and lower deviations between the methods.

**Figure 5:**
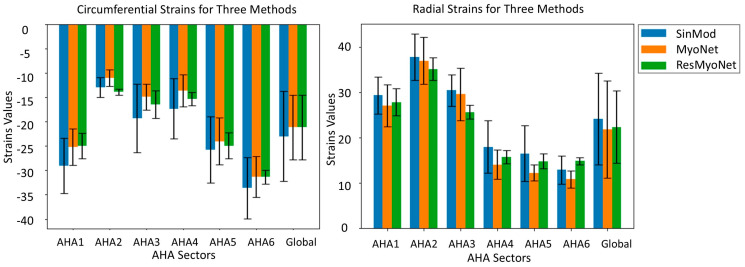
Bar plots illustrate circumferential (Ecc) and radial (Err) strains from three imaging methods: SinMod, MyoNet, and ResMyoNet, across the six AHA-defined segments with added global strain values.

Figure 4 displays the x- and y-displacement fields obtained from SinMod, MyoNet, and ResMyoNet. The accompanying error maps provide insight into the differences between these displacement fields, underscoring the differences and potential implications for the fidelity and accuracy of each approach in evaluating myocardial displacements. Following this, bar plots in Figure 5 were generated to depict the circumferential (Ecc) and radial (Err) strains obtained through three distinct imaging techniques: tagging (gold standard), MyoNet, and ResMyoNet. These measurements were specifically performed for each of the six segments per slice as defined by the American Heart Association (AHA) segmentation model [34]. These bar plots by the three methods (SinMod-derived reference, MyoNet, and ResMyoNet) show remarkably analogous patterns. In addition, a global analysis was conducted to provide a comprehensive assessment of the strain metrics across all segments. Both Ecc and Err for each of six segments per slice underwent statistical analysis using Student’s *t*-test with Bonferroni correction for multiple comparisons. No segments showed a statistically significant difference after correction (*p* < 0.05). Without correction, one segment (anterior) in Err from MyoNet and one segment (inferoseptal) in Err from ResMyoNet were statistically different when compared to SinMod-derived values (Table 1).

**Figure 6:**
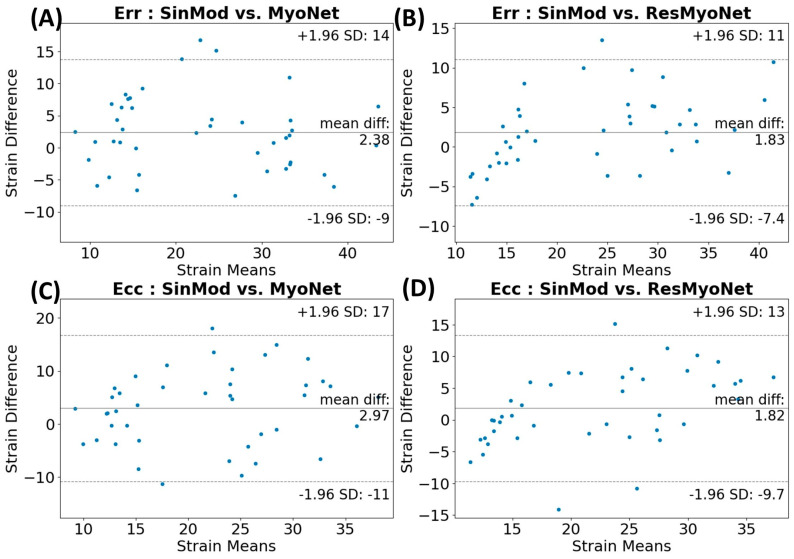
Bland–Altman plots comparing strain measures for (**A**) Ecc: SinMod vs. MyoNet, (**B**) Ecc: SinMod vs. ResMyoNet, (**C**) Err: SinMod vs. MyoNet, and (**D**) Err: SinMod vs. ResMyoNet. Across both Ecc and Err, the majority of data points fall within the 2 standard deviation limits, indicating a high degree of agreement between the methods. Each data point represents a segment, with a total of 42 segments (from 7 test datasets, each yielding 6 segments included in these plots).

Bland–Altman plots were constructed (Figure 6) to compare the strain measures between SinMod vs. MyoNet and SinMod vs. ResMyoNet. Bland–Altman analysis demonstrated minimal systematic bias between methods. For Ecc, MyoNet showed a mean bias of −1.9%, while ResMyoNet showed a mean bias of −2.0%. For Err, MyoNet demonstrated a mean bias of 1.0%, while ResMyoNet showed a mean bias of 2.0%. These small biases suggest clinically acceptable agreement between deep learning methods and the SinMod reference. This consistency is especially noteworthy as the data points encompass all segments from the test dataset.

### 3.2. Model Performance and Computational Efficiency

**Figure 7:**
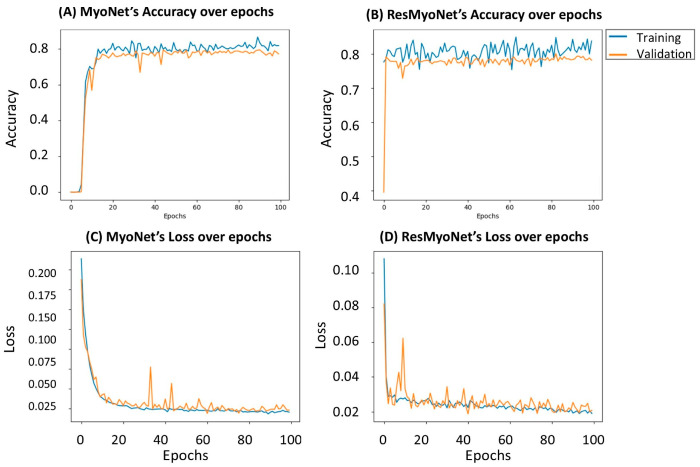
Evaluation of MyoNet and ResMyoNet performance over training epochs. (**A**) Accuracy of MyoNet, (**B**) Accuracy of ResMyoNet, (**C**) Loss of MyoNet, and (**D**) Loss of ResMyoNet. ResMyoNet quickly achieves high accuracy and maintains low loss values, whereas MyoNet takes more epochs to reach comparable levels.

The performance consistency of both MyoNet and ResMyoNet networks was further assessed through an analysis of accuracy and loss over the span of the training epochs. As demonstrated in Figure 7, while both models underwent training for 100 epochs, ResMyoNet notably reached high and stable accuracy levels along with low and consistent loss values within just a few initial epochs. This rapid stabilization contrasts with MyoNet, which required more epochs to achieve similar performance metrics.

A comparative analysis was conducted to discern the efficiency of the MyoNet and ResMyoNet in the context of training, characterized by evaluating their computation times across 10 and 100 epochs. MyoNet required a computation time of 158 s for 10 epochs, escalating to 1546 s for 100 epochs. In contrast, ResMyoNet requires only 124 s for 10 epochs and 1177 s for 100 epochs. For all measurements, ResMyoNet consistently exhibited shorter computation durations when compared to MyoNet, emphasizing its relative efficiency in the training phase.

### 3.3. Performance Metrics of MyoNet and ResMyoNet

To assess and compare the performance of the MyoNet and ResMyoNet networks for generating Ecc and Err, we utilized multiple metrics (Table 2). For displacement field accuracy, MyoNet achieved RMSE of 1.06 mm in x and 0.94 mm in y-displacement fields, while ResMyoNet achieved RMSE of 1.24 mm in x and 1.00 mm in y-displacement fields. Examining the SSIM values, MyoNet achieved higher values (0.961, 0.960) compared to ResMyoNet (0.937, 0.934). As for ICC, MyoNet displayed slightly higher than ResMyoNet. For the Pearson CC analysis, both networks showed strong correlations. Lastly, concerning the CV metrics, MyoNet’s values were presented as higher than ResMyoNet’s.

## 4. Discussion

We demonstrate that the developed MyoNet and ResMyoNet each show unique characteristics and differ in their performance metrics, underscoring the distinct capabilities and strengths of each network.

In contrast to existing methodologies [35], which focused on mice with a small cohort size (*n* = 8), the proposed algorithm is validated on a larger and more diverse dataset. This enhances the generalizability of the results. Moreover, while the approach in [35] requires manual localization of the LV blood pool center point, it automates this step, thereby reducing potential sources of human error and increasing efficiency. The Siamese architecture’s bidirectional motion learning [36] offers a dual pathway for processing input data, allowing the simultaneous analysis of different frames. This approach ensures that the temporal relationships between consecutive frames are well = established, fostering a more precise capture of myocardial motion dynamics. However, bidirectional motion learning may overlook subtle myocardial motion patterns that do not manifest across both pathways, and this might make the model more sensitive to irregularities or noise in input data sequences.

The use of the 3D convolutional approach (spatiotemporal) over the 2D (spatial) with subsequent 1D (temporal) strategy (2D + 1D) [37] is multifaceted. The 3D convolution inherently integrates spatial and temporal information in a unified manner, capturing the intricate relationships across three dimensions. In contrast, the 2D + 1D approach would first process spatial data and subsequently add the temporal dimension, potentially missing interactions between spatial and temporal features that are crucial for myocardial strain analysis. Importantly, the proposed networks alternate kernel sizes that focus on the spatial and temporal dimensions alternatively. MyoNet and ResMyoNet, with their unique designs, emphasize the proficient extraction of both spatial and temporal features, illustrated by kernel size alternations. Traditional methodologies (non-alternating kernel size) often grapple with the complexity of parsing data that spans both spatial and temporal dimensions. To address this, the proposed networks leverage deep learning’s inherent strengths in processing spatial and temporal information synergistically, aiming to augment the accuracy and efficiency of strain analysis in cardiac imaging. This indicates a dedicated mechanism for assimilating spatial and temporal variations within the input data. Moreover, the convolutional operations employ dilation, expanding the receptive field without compromising spatial resolution. This has the capability to capture extensive spatiotemporal patterns, possibly elucidating the superior accuracy metrics associated with MyoNet and ResMyoNet.

ResMyoNet integrates the potency of residual connections, known for circumventing the vanishing gradient problem, ensuring efficient backpropagation even in deeper architectures [28]. This feature is likely pivotal in facilitating a rapid training convergence for ResMyoNet, a probable explanation for its reduced training computational time. Yet, it is paramount to acknowledge inherent constraints. While MyoNet’s focus on detail improves accuracy, it concurrently demands more computational resources. ResMyoNet, although optimized for efficiency, might present a subtle compromise on intricate detail extraction in certain cases. These tradeoffs underscore the balance between accuracy and efficiency in deep learning models. If optimized accuracy and attention to detail are the priority, then MyoNet is the preferred choice for the user. However, when rapid training and computational efficiency are crucial, ResMyoNet is the more appropriate choice.

The selection of loss functions is crucial to balance accuracy with robustness. The mean squared error (MSE) ensures a foundational penalty for discrepancies in predicted x- and y-displacement fields. However, to address potential outliers such as anomalous displacement fields, inaccurate predictions, and data acquisition inconsistencies, the smooth L1 loss was employed. This loss transitions between penalizing small and large discrepancies, enhancing model robustness. Furthermore, the introduction of a custom loss function focuses on regions of pronounced cardiac strain. By emphasizing errors in segmented displacement areas and setting thresholds for significant displacements, this custom loss function ensures that the networks prioritize myocardial contractility patterns, merging precision with specificity.

Our study has some limitations. First, while there are minimal variations in the imaging parameters between cine and tagging sequences, it is important to note that both sequences capture the same spatial (slice) and temporal (timeframes) dimensions. This can potentially manifest as systematic biases in the strain evaluations determined by our developed models, thereby influencing the direct comparability of results derived from both sequences. Notably, the results illustrated that, in the context of Err strains, MyoNet’s interpretation of the anterior segments and ResMyoNet’s interpretation of the septal segments showed statistical differences when compared to tagging. This divergence might indicate that certain myocardial segments may be more susceptible to variations in the imaging sequences for different networks, or there might be differences between these specific segments that the networks process differently. Addressing such specific inconsistencies is important for the practical implementation of deep learning techniques in myocardial strain analysis. Another limitation is that our dataset was limited to 64 cases, each composed of 20 timeframes. While this dataset has been instrumental for drawing preliminary conclusions, the robustness and generalizability of the findings would undoubtedly benefit from a larger dataset. Another limitation of the proposed algorithms is that they focus on analyzing short-axis images, providing circumferential and radial strains. Future work includes generalizing algorithms to include analysis of long-axis slices. The demonstrated efficacy and insights provided by both MyoNet and ResMyoNet signify a promising step forward for pre-clinical cardiac imaging and strain analysis. Their capabilities, particularly in accurately interpreting spatiotemporal data, could offer researchers a more comprehensive and in-depth understanding of myocardial contractility patterns, potentially improving personalized diagnosis and treatment strategies. MyoNet provides superior quantitative metrics, while ResMyoNet offers a more efficient network. As these networks undergo further refinement and are validated on larger, more diverse datasets, translation to clinical practices becomes increasingly promising, marking a significant advancement in artificial intelligence (AI)-augmented cardiac care.

## 5. Conclusions

Our study demonstrates the significant advancements MyoNet brings to myocardial strain analysis in cine CMR, offering a notable improvement over the traditional tagging methods. Its innovative DL architecture enables precise and efficient analysis of myocardial strain directly from cine images, thereby reducing the need for tagged acquisitions and shortening acquisition times. This progress not only makes CMR more cost-effective in research and pre-clinical settings but also minimizes patient discomfort and scan times, expanding the utility of CMR in research applications. While ResMyoNet also contributes to the field in terms of training efficiency, MyoNet stands out for its comprehensive analysis capabilities. As MyoNet and ResMyoNet continue to evolve, validating them with comprehensive patient datasets will be crucial for their future clinical translation.

## Figures and Tables

**Table 1:** t-statistic and *p*-value results from Student’s *t*-test.

	MyoNet		ResMyoNet	
	Ecc	Err	Ecc	Err
AHA 1	t = 1.45	t = 3.16	t = 1.80	t = 1.42
	*p* = 0.20	*p* = 0.02 *	*p* = 0.12	*p* = 0.20
AHA 2	t = 1.65	t = 0.37	t = 0.96	t = 1.55
	*p* = 0.15	*p* = 0.72	*p* = 0.38	*p* = 0.17
AHA 3	t = 1.89	t = 0.30	t = 1.34	t = 2.88
	*p* = 0.11	*p* = 0.77	*p* = 0.46	*p* = 0.03 *
AHA 4	t = 1.14	t = 1.28	t = 0.79	t = 1.05
	*p* = 0.30	*p* = 0.25	*p* = 0.80	*p* = 0.49
AHA 5	t = 0.46	t = 1.58	t = 0.26	t = 0.74
	*p* = 0.66	*p* = 0.17	*p* = 0.80	*p* = 0.49
AHA 6	t = 0.75	t = 1.74	t = 0.95	t = −1.53
	*p* = 0.48	*p* = 0.13	*p* = 0.38	*p* = 0.18

*: significant difference. AHA, American Heart Association.

**Table 2:** Comparative performance metrics of MyoNet and ResMyoNet in generating Ecc and Err.

	MyoNet		ResMyoNet	
	Ecc	Err	Ecc	Err
SSIM	0.961	0.960	0.937	0.934
ICC	0.973	0.975	0.955	0.955
Pearson CC	0.973	0.975	0.956	0.955
CV	32.447	34.445	21.749	22.116

SSIM, structural similarity index; ICC, intraclass correlation coefficient; Pearson CC, Pearson correlation coefficient; CV, coefficient of variation.

## Data Availability

Restrictions apply to the datasets. The datasets used in this study are available from the corresponding author upon reasonable request, subject to institutional approval. Code can be shared with editors and reviewers upon reasonable request.

## Abbreviations

The following abbreviations are used in this manuscript:

DL	Deep learning
CMR	Cardiac magnetic resonance
RT	Radiation therapy
Ecc	Circumferential strain
Err	Radial strain
LVEF	Left ventricular ejection fraction
SS	Salt-sensitive
CMR-FT	CMR feature tracking
LV	Left ventricle
RMSE	Root mean squared error
MSE	Mean squared error
SSIM	Structural similarity index
ICC	Intraclass correlation coefficient
CV	Coefficient of variation
AHA	American Heart Association
SD	Standard deviation
